# Sick sinus syndrome as the initial manifestation of neuromyelitis optica spectrum disorder: a case report

**DOI:** 10.1186/s12883-022-02580-x

**Published:** 2022-02-14

**Authors:** Mai Hamaguchi, Hiroaki Fujita, Tomonari Suzuki, Keisuke Suzuki

**Affiliations:** 1 Department of Neurology, Dokkyo Medical University, 880 Kitakobayashi, Mibu, Shimotsuga, Tochigi, 321-0293 Japan; 2 Clinical Training Center, Dokkyo Medical University Hospital, Tochigi, Japan

**Keywords:** Neuromyelitis optica spectrum disorder, Medulla lesion, Sick sinus syndrome, Area postrema, Case report

## Abstract

**Background:**

Sick sinus syndrome (SSS) is known to occur due to lesions in the medulla oblongata. Although medullary lesions have occurred in patients with neuromyelitis optica spectrum disorder (NMOSD), there are few reports of SSS associated with NMOSD. We report a patient with NMOSD who developed refractory nausea, vomiting and SSS as the initial manifestation.

**Case presentation:**

A 77-year-old female developed refractory nausea and frequent episodes of syncope. The patient was diagnosed with SSS because sinus pauses lasting five to six seconds were observed, and pacemaker implantation was performed. Two months later, she was referred to our hospital because of limb weakness and sensory impairment that progressed over a month. The patient was confirmed to have muscle weakness; manual muscle testing revealed grade 4 in the upper extremities and grade 3 in the lower extremities. Tendon reflexes were diminished, while no pathological reflexes were present. Thermal and pain sensations were impaired in the upper and lower extremities, and vibration sensation was impaired in both lower extremities. Bladder and rectal disturbances were also noted. Optic neuritis was not detected. T2-weighted magnetic resonance imaging (MRI) showed high-intensity lesions in the dorsal part of the medulla oblongata and C3–6 cervical cord. Her serum was positive for antibodies against aquaporin 4, and a diagnosis of NMOSD was made. She was treated with two courses of an intravenous methylprednisolone pulse and one course of plasma exchange. Then, she was transferred to another hospital for rehabilitation.

**Conclusions:**

Because SSS is a life-threatening complication, clinicians should be aware of the possibility that medullary lesions in NMOSD can cause SSS as the initial manifestation.

## Background

Neuromyelitis optica (NMO) is an autoimmune inflammatory astrocytopathy of the central nervous system that leads to secondary oligodendrocyte loss and is characterized by optic neuritis and extensive transverse myelitis [1]. After the identification of anti-aquaporin 4 (AQP4) antibodies and recognition of disease-related brain lesions in the area postrema, brainstem and diencephalon, the concept of NMO has been expanded to NMO spectrum disorder (NMOSD) [2]. There are some reports of sick sinus syndrome (SSS) caused by lesions in the medulla oblongata [3]. Potentially fatal bradyarrhythmia can occur in patients with NMOSD involving the area postrema, but there are few reports of SSS associated with NMOSD [4–8]. Here, we report a patient with NMOSD who developed refractory nausea, vomiting and SSS as the initial manifestation.

## Case presentation

**Fig. 1:**
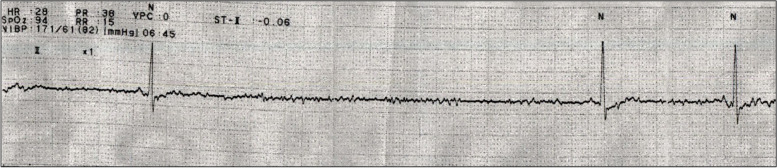
Electrocardiogram recorded before pacemaker implantation. The electrocardiogram recorded before pacemaker implantation showed sinus pauses that continued for five to six seconds

**Fig. 2:**
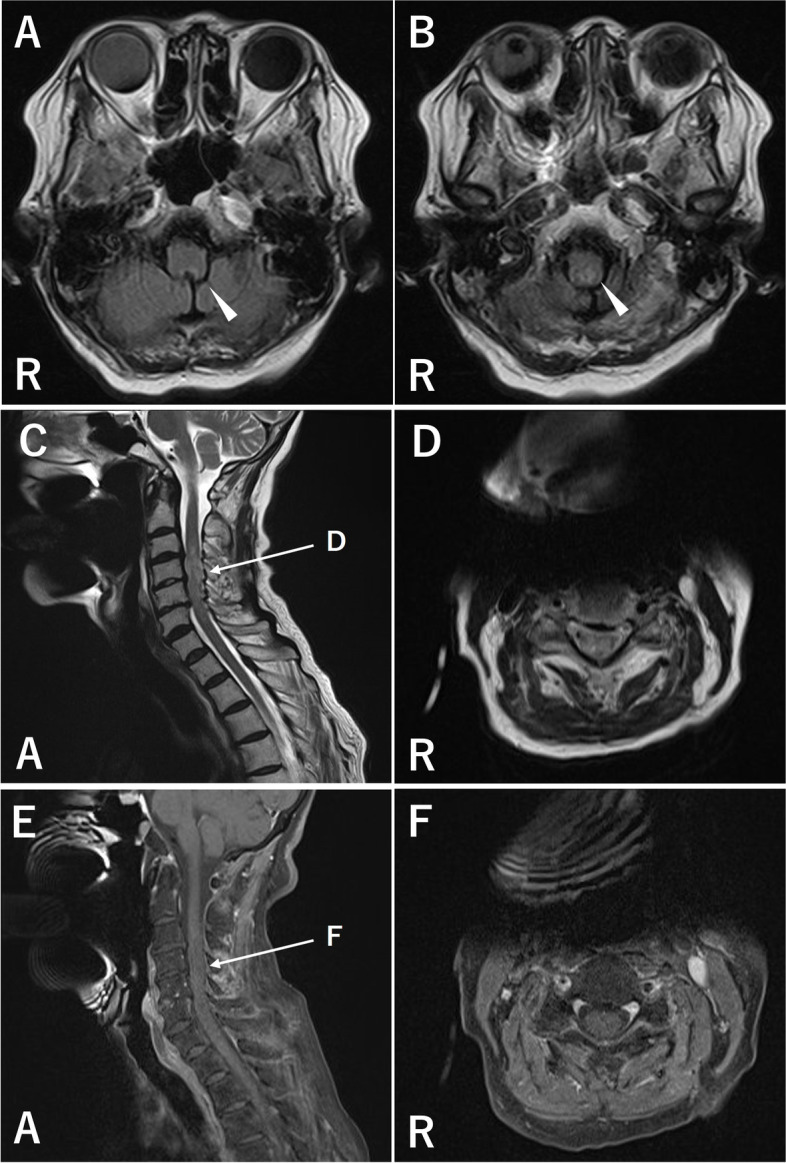
MR imaging of the brain and cervical cord. Axial FLAIR brain MRI revealed a high-intensity lesion in the dorsal part of the medulla oblongata (**A**, **B**; arrowheads). On T2-weighted cervical MRI, a high-intensity lesion existed in the C3–6 cervical cord (**C**, **D**), while slight enhancement was observed in those corresponding areas on gadolinium-enhanced T1-weighted images (**E**, **F**)

A 77-year-old female was admitted to a previous hospital with refractory nausea and vomiting. She showed several episodes of syncope, and electrocardiogram (ECG) monitoring indicated sinus pauses lasting five to six seconds **(**Fig. 1**)**, and permanent pacemaker implantation was performed after the diagnosis of SSS. Two months after the operation, she was referred to our hospital because of weakness of the upper and lower extremities and sensory impairment of the lower extremities, which progressed over a month. She had 10-year history of type 2 diabetes mellitus, hypertension and dyslipidemia but had no history of cardiac disease before pacemaker implantation. Her consciousness was intact, and there was no abnormality in the cranial nerves. The patient had muscle weakness; manual muscle testing revealed grade 4 in the upper extremities and grade 3 in the lower extremities. Tendon reflexes were diminished with no pathological reflexes. Thermal and pain sensations were impaired in the upper and lower extremities, and vibration sensation was impaired in both lower extremities. Bladder and rectal disturbances were also noted. Electrocardiography revealed a pacemaker rhythm, and cardiac ultrasonography showed no abnormalities. On laboratory examination, serum anti-AQP4 antibody was positive. The soluble interleukin 2 receptor level was 638 U/mL. Anti-nucleotide antibody, ACE, anti-SS-A antibody, anti-SS-B antibody, PR3-ANCA, MPO-ANCA and anti-TPO antibody were not elevated. The plasma glucose level was 137 mg/dL, HbA1c was 7.0%, the CK level was 25 U/L, and the BNP level was 131.9 pg/mL. CSF examination showed elevated protein levels with normal cell counts. The IgG index was 0.47, and the myelin basic protein level was not elevated. Oligoclonal IgG bands were negative. On fluid-attenuated inversion recovery (FLAIR) brain magnetic resonance imaging (MRI), a high-intensity lesion was found in the dorsal part of the medulla oblongata without enhancement, but no lesion was found in the hypothalamic or supratentorial region. T2-weighted whole spinal cord MRI showed a high-intensity lesion at cervical levels 3–6, with faint enhancement on gadolinium-enhanced T1-weighted imaging **(**Fig. 2**)**.

Optic neuritis was not detected on ophthalmologic examination. The patient was diagnosed with NMOSD and treated with two courses of an intravenous methylprednisolone pulse and one course of plasma exchange. Although symptoms of area postrema, such as nausea, improved after treatment, the patient remained chair bound and had residual bowel/bladder symptoms, and on 30 mg oral prednisolone with a pacemaker. Her physical activity was deteriorated because of disuse. Because she refused any further treatment, no additional treatment was performed. She was transferred to another hospital for rehabilitation.

## Discussion and conclusions

We report a patient with NMOSD with dorsal medulla and cervical cord lesions who initially presented with area postrema syndrome (that is, refractory nausea and vomiting) and potentially fatal bradycardia and subsequently required pacemaker implantation based on a diagnosis of SSS. Area postrema syndrome associated with medullary lesions is known to occur in patients with NMOSD [2, 9]. However, the possibility that dorsal lesions of the medulla oblongata in NMOSD may also produce SSS has not received much attention. There are some reports of arrythmia caused by medullary lesions, such as multiple sclerosis, [10, 11] sarcoidosis [12], and Wallenberg syndrome [13]. Medullary lesions caused by multiple sclerosis are also accompanied by takotsubo cardiomyopathy [14] and orthostatic hypotension [15]. However, only a few patients with NMOSD who presented with SSS due to medullary lesions have been reported. Table 1 summarizes the six previously reported NMOSD patients presenting SSS associated with medullary lesions, including our patient [4–8]. In our patient, frequent episodes of syncope were among the initial symptoms.

**Table 1:** Patients with neuromyelitis optica spectrum disorder presenting with arrhythmia

Author, year (ref)	Age (years), sex	Type of arrhythmia	Other symptoms	MRI lesion	Treatment	Outcome
Tsouris et al., 2020 [6]	42, M	Sinus pauses	Hiccups, reduced visual acuity of the left eye, retrobulbar pain	Lower medulla oblongata, left optic nerve	PMI, IVMP and oral PSL	Complete recovery
Endo et al., 2020 [5]	22, F	Sinus arrest	Nausea, vertigo, nystagmus, diplopia, dysarthria, paresthesia of the left limbs	Dorsal part of the medulla oblongata	Temporary PM, IVMP and oral PSL	Improved
Komaki et al., 2020 [4]	77, M	Sinus arrest	Hiccups, vomiting	Area postrema	Temporary PM and oral PSL	Improved
Okada et al., 2012 [7]	78, M	Cardiorespiratory arrest	Hiccups, nausea, orthostatic hypotension, dysarthria, dysphagia	Medulla oblongata, cervical cord	IVMP, acyclovir, IVIg	Partially improved
Bigi et al., 2012 [8]	16, M	Sinus bradycardia	Hiccups, vomiting	Area postrema	IVMP	Complete recovery
Our patient	77, F	Sinus pauses	Nausea, vomiting, weakness of extremities, sensory impairment of the lower extremities	Dorsal part of the medulla oblongata, cervical cord	PMI, IVMP, PE and oral PSL	Partially improved

*IVMP* intravenous methylprednisolone, *PE* plasma exchange, *PMI* pacemaker implantation, *PSL* prednisolone

The patients reported by Tsouris and Okada et al. [6, 7] were admitted with a chief complaint of syncope, while syncope was noticed early after admission in the patients reported by Endo and Komaki et al [4, 5]. Bigi et al. [8] reported a young patient with area postrema involvement who displayed vomiting, hiccups, and sinus bradycardia. Notably, all the patients had lesions in the dorsal or lower part of the medulla oblongata and exhibited area postrema syndrome (nausea, vomiting and hiccups), which indicates that SSS and area postrema syndrome are caused by lesions in close proximity. Therefore, it is reasonable to consider that our patient’s medullary lesion caused both area postrema syndrome and SSS as the initial presentation, although MRI imaging was not immediately performed after the onset of symptoms. Temporary or permanent pacemakers were required in four of six patients because of recurrent episodes of syncope caused by SSS. After immune treatment, two of the four patients were weaned off pacing support.

SSS associated with medulla oblongata or cervical cord lesions has been previously reported [3–8, 16]. It is presumed that damage to the solitary nuclei of the medulla oblongata stimulates the vagal nerve, causing bradycardia [3]. On the other hand, Profice et al. [16] reported that a 35-year-old female with C2 cervical myelitis of unknown etiology developed sudden-onset sinus bradycardia. Decreased uptake of cardiac metaiodobenzylguanidine (MIBG) scintigraphy in that patient suggested that her bradyarrythmia may have been caused by parasympathetic predominance due to impairment of the spinal sympathetic nerve descending from the brainstem to the Th1-L2 intermediolateral nucleus. In our patient, one month had elapsed between the onset of area postrema syndrome with SSS due to the medullary lesion and the onset of quadriplegia and sensory deficit due to the cervical cord lesion. In NMOSD patients with a relapsing course, approximately 60% relapse within one year, and 90% relapse within three years. Repetitive recurrences make the disease prognosis worse because of the accumulation of neurological damage and sequelae [17]; therefore, earlier diagnosis and treatment may improve the prognosis of patients. Furthermore, the patient presented here was much older than the typical NMOSD patient, which made the diagnosis even more difficult.

It is necessary to monitor the ECG of NMOSD patients with medulla oblongata lesions because SSS is a life-threatening complication. Area postrema syndrome, including potentially fatal bradycardia, can occur in cases of NMOSD with medullary lesions. We should be aware of the possibility that medullary lesions in NMOSD can cause SSS as the initial manifestation. For patients with area postrema syndromes or medullary lesions caused by NMOSD, multiple sclerosis or other conditions, ECG monitoring for at least several days is necessary.

## Data Availability

All data generated or analysed during this study are included in this published article.

## Abbreviations

NMO: Neuromyelitis optica
AQP4: Aquaporin 4
NMOSD: NMO spectrum disorder
SSS: Sick sinus syndrome
ECG: Electrocardiogram
FLAIR: Fluid attenuated inversion recovery
